# The Relationship Between Factors Related to Divorce Request and Mental Health Among Divorce Applicant Women Referred to Legal Medicine Organization in Ahvaz, Iran 

**Published:** 2017-09

**Authors:** Lida Jalili, Shahnaz Najar, Salimeh Nezamivand-Chegini, Masoumeh Yaralizadeh

**Affiliations:** 1 Reproductive Health Promotion Research Center, Midwifery Department, Ahvaz Jundishapur University of Medical Sciences, Ahvaz, Iran; 2 Midwifery Department, Ahvaz Jundishapur University of Medical Sciences, Ahvaz, Iran; 3 Midwifery Department, Tabriz University of Medical Sciences, Tabriz, Iran

**Keywords:** Divorce Applicant Women, Mental Health, Factors Affecting Divorce, Questionnaire Scl-25

## Abstract

**Objective:** This study aimed to investigate the relationship between factors related to divorce request with mental health among divorce applicant women in order to understand the effect of these factors on women's mental health.

**Materials and methods:** This study was a cross-sectional study performed on 434 divorce applicant women who referred to legal medicine department of Ahvaz in 2013 based on convenience sampling. Information was collected by using researcher made questionnaire for factors affecting divorce and symptom checklist-25 (SCL-25) standard questionnaire. The data were analyzed using SPSS ver.18.

**Results:** The results showed that among the social factors, life skills and communication, family and individual factors had a significant relationship with mental health among divorce applicant women (p < 0.05). No relationship was seen with economic and cultural factors affecting divorce request (p > 0.05).

**Conclusion:** Regarding the negative effects of various causative factors of divorce on mental health of women including social, life skills, communication, family and individual factors strategies for prevention and reduction of these factors should be seriously considered for prevention and early treatment of mental health problems. These strategies include counseling before marriage, after marriage and during the divorce process.

## Introduction

Family is the oldest social organization that provides an important role in the mental health of the community. Disruption or failure of the family can be defined as the breakdown of family unitor the failure to build social roles due to the failure or inability of one or more family members in performing their roles (1). Psychologists believe that the health of the family can be achieved by establishing a strong and permanent link between woman and man (2). Therefore, divorce phenomenon is among the problems of concern in all communities is the (3). Divorce starts with the experience of emotional crisis between couples and ends by making effort to solve the conflict through separation and entering into a new position with a new set of roles and lifestyle (4). Often, one of the couples endures a heavier dependence, conflict and emotional pain than the other for a longer duration (5).In most cases women are more exposed to the destructive effects of divorce than men (5).

Divorce rate is increasing in various countries, including the Netherlands (6) and Canada (7). According to official statistics in Iran, 200 cases of divorce occur out of every one thousand marriages, which has made Iran the fourth country in the world with high divorce rate (8). According to the Statistical Yearbook of the country in Khuzestan province 5.8% of marriages ended up in divorce (3198 cases of divorce occurred in 37516 of marriages) while in 2012 this figure increased to 11.66% (9).

Several factors may affect mental health of individuals. Numerous studies have shown that many health problems and mental disorders have social roots. Divorce can possess similar effects on public health as it does on family life (10). Divorce leads to mental and emotional imbalance in individuals. Divorce, as a severe stress, can cause psychiatric disorders, particularly in women, who are characterized by their emotional delicacy. Unfortunately, mental health problems have become one of the most important social dilemmas with increasing divorce rate (11).

In Australia Hewitt and colleagues (2010) showed that unlike women's physical health, which improves after divorce, their mental health is reduced. They also found that the consequences of divorce are less severe in individuals who apply for divorce themselves and in cases that chose divorce by mutual consent (12). Another study by Cohen and colleagues (2012) in Israel showed a significant association between mental health and some of the causes of divorce and external factors affecting divorce (13). Moreover, Barons study (1989) in Nebraska showed that the reasons for divorce can predict self-esteem and psychological distress after divorce (14).

According to the effect of mental health of women in the family and the important role of family in society and given that few studies have assessed the relationship between mental health and factors affecting divorce, this study was designed to investigation the relationship between factors affecting divorce request and mental health among divorce applicant women who referred to Ahvaz Legal Medicine department, in 2013.

## Materials and methods

This cross-sectional study was performed on 434 divorce applicant women who referred to the Ahvaz legal medicine department based on convenience sampling in 2013 years. The sample size of 434 subjects was calculated based on a study (15) and also by using NCSS software considering 90% power and 95% confidence level.Allof women seeking divorce who were referred to the Ahvaz Legal Medicine department to perform pregnancy testand agreed to participate in the study were included in the study. Exclusion criteria were known history of psychiatric disease before marriage, consumption of medications that affect mental health (tricyclic antidepressants, chlordiazepoxide, etc.) and history of a traumatic event such as the death of relatives in the past six months.

Status and factors related to divorce were assessed using a questionnaire that included seven parts: 1) personal information including age, education, income, family characteristics of individuals, how the partner was chosen and familiarity with partner, type of marriage- mandatory or voluntarily, divorce status in family, etc., 2) Social factors of divorce including addiction, delinquency and criminal convictions, 3) Cultural factors including opposition to employment and education of partner, adherence to superstitious ideas, disagreements in parenting, 4) Economic factors including unemployment, lack of living facilities, 5) Life skills and communication related factors (lack of love, lack of respect for partner or family), 6) Individual factors including lack of adornment, physical abuse and insults, 7) Family factors including dispute, opposition to family trips, living with family and at the end of the questionnaire an open question asked for the other factors that played a role in divorce. The questionnaire was completed from the perspective of the woman about her husband and divorce. This questionnaire has been assessed for face and content validity (16). To assess the content validity the questions were designed based on the review of literature for both national and international; studies that defined the factors that influence on decision to divorce. In the next step, the questionnaire was reviewed by experts and then final changes were made to the questions, the reliability of the questionnaire was assessed based on test-retest method (alpha = 0.81)

Data from questionnaires included seven factors affecting divorce (demographic, social, cultural, economic, and life skills related to communication, personal and family factors for divorce) and a section including factors that women themselves proposed. In order to evaluate the mental health of women symptom checklist-25 (SCL-25) questionnaire was used.

SCL-25 is a single-factor scale questionnaire to measure mental health. This scale is a summary of the symptom checklist-90 (SCL-90) designed by David and Najarian. In a study performed by Asgari et al. (2008), SCL-25 questionnaire was validated against the criterion (general health Goldenberg), and a significant relationship was observed between mental health questionnaire and the criterion (p = 0.0001, r = 90.5) (15). The scale reliability coefficients were calculated using Cronbach's coefficients (0.92) and Spearman- Brown (0.88) and Guttman (0.87), which represents a good reliability (15). The questionnaire was filled by researcher for each subject after explaining the aims of study, ensuring the integrity of the information obtained from them and obtaining consent.

The data were analyzed using the statistical package for social sciences (SPSS) software ver.18. To analyze the data, descriptive and inferential statistics were used. Descriptive statistics were used to determine the average and standard deviation and to adjust the relative frequency distribution tableand inferential statistics. The Kolmogorov -Smirnov test was used to assess the normality of continuous variables at α = 0.05. For between groups comparison of normally distributed variables the one-way analysis of variance (ANOVA) and Pearson correlation coefficient and independent t-test were used while the Mann-Whitney test was used to compare non-normally distributed variables between groups. Generalized linear model (GLM) analysis was used to assess the relationship between study parameters and mental health scores after adjustment for confounding variables. The value of p < 0.05 was considered statistically significant.

## Results

A total of 434participants completed the questionnaire and were included in the final analysis. Of the 434 women enrolled in the study, 238 (54.8%) were of Arab ethnicity, 41 (9.4%) were younger than 20 years old, 196 (45.2 %) had no children. In 46.1 % of subjects divorce occurred in the first five years of marriage. The divorce rate among working women (1.4%) was lesser than the other groups. Monthly income of 361 subjects (83.2%) was below 500 united States Dollars (USD), 267 (61.5%) of subjects reported a parental households of more than 4 people. Using one-way ANOVA a significant different was observed in SCL-25 score between the levels of education (p=0.002) and job categories (p = 0.022) while t-test showed a significant difference in SCL-25 scores between number of households (p = 0.001) (Table 1).

**Table 1:** Distribution of demographic characteristics of divorce applicant women and its relation to mental health

**Characteristics**		**n (%)**	**Mental Health**	P-value*
			**Mean (SD)**	
Age (year)	< 20	41 (9.4)	18.7 (33.2)	0.232
	20-30	212 (48.8)	38.1 (21.8)	
	30-40	121 (27.9)	37.5 (22.3)	
	> 40	13.8 (60)	32.7 (18.7)	
Education level	Primary/Illiterate	108 (24.9)	39.4 (22.2)	0.002
	Guidance/High school	234 (53.9)	38.5 (21.4)	
	College	92 (21.2)	39.0 (23.5)	
Children numerous	No children	196 (45.2)	36.6 (21.2)	0.900
	1-3	217 (50)	36.8 (22.2)	
	> 3	21 (4.8)	38.8 (19.0)	
Number of main household	< 4	166 (38.2)	32.6 (19.7)	0.001
	> 4	268 (61.5)	39.3 (21.9)	
Length of common life	< 5	200 (46.1)	36.3 (21,3)	0.429
	6-10	100 (23)	38.8 (20.5)	
	11-15	52 (12)	38.8 (22.4)	
	>15	82 (18.9)	34.1 (21.6)	
Occupation	Housewife	321 (74)	36.9 (21.6)	0.022
	Self-employment	46 (10.6)	44.8 (31.0)	
	Employee	7 (15.5)	36.8 (19)	
Nationality	Arab	238 (54.8)	21.4 (37.6)	0.426
	Bakhtiyari/Lor	129 (29.7)	21.7 (36.9)	
	Other	67 (15.4)	20.3 (33.7)	
Income	Less than 500	361 (83.2)	37.1 (21.5)	
	500-1milion	63 (14.5)	34.9 (21.3)	
	More than 1milion	10 (2.3)	35.6 (16.2)	0.740

**Table 2:** Relation between factors affecting in divorce with mental health in divorce applicant women

**Factors affecting divorce**	**Mental Health**						**p.value**
	**Less than 18**			**More than 18**			
	**Second ** **quarter**	**First ** **quarter**	**Median**	**Second ** **quarter**	**First ** **quarter**	**Median**	
Social factors	6	0	2	6	0	3	0.017
Cultural factors	9	2	7	9	2	6	0.316
Economic factors	6	0	4	6	0	4	0.541
Life skills and communication factors	24	9.2	17	24	10	16	0.267
Family factors	16	6	12	16	5	11	0.291
Individual factors	7	2	14	7	2	5	0.044

Generalized linear model with adjustment for confounding variables was used to test the study hypotheses. GLM revealed that mental health scores were significantly associated with positive response to social factors (p = 0.022) and individual factors (p = 0.002). Mann-Whitney test showed a significant difference in SCL-25 scores between the social factors (p = 0.017) and personal factors (p = 0.045) (Table 2).

Among the factors affecting divorce that subjects blamed themselves of "indifference to sexual relations" (140 subjects, 32.3%) was the most frequent while "Woman's physical or mental illness"(in 18 subjects, 1.4%) was the least frequent reason. Chi-square test revealed a significant association between mental health score category and the above mentioned factors (Table 3).

**Table 3:** The relationship between mental health and factors that women themselves were blamed on them

Factors that women blame on its own		Mental Health			p.value
		Less than 18	More than 18	Total	
Excuse-making, Women reproach	Yes	23 (21.1)	104 (32)	127 (29.3)	0.019
	No	86 (78.9)	221 (68)	307 (70.7)	
Suspicion and mistrust woman	Yes	19 (17.4)	101 (31.1)	120 (27.6)	0.003
	No	90 (82.6)	224 (68.9)	314 (72.4)	
Impatient and angry woman	Yes	23 (21.3)	115 (35.4)	139 (32)	0.004
	No	85 (87.7)	210 (64.6)	295 (68)	
Lack of consultation in life by women	Yes	10 (9.2)	56 (17.2)	97 (22.4)	0.027
	No	99 (90.8)	269 (82.8)	337 (77.6)	
Embarrassment, fear or duress in sexual relations	Yes	13 (11.9)	72 (22.2)	85 (19.6)	0.012
	No	96 (88.1)	253 (77.8)	349 (80.4)	
Female sexual problems	Yes	11 (10.1)	76 (23.4)	87 (20.05)	0.001
	No	98 (89.9)	249 (76.6)	347 (79.9)	
Indifference to sexual relations	Yes	25 (22.9)	115 (35.4)	140 (32.3)	0.010
	No	84 (77.1)	210 (64.6)	294 (67.7)	
Disrespect to the family man	Yes	7 (6.4)	69 (21.2)	76 (17.5)	0.000
	No	102 (93.6)	256 (78.8)	358 (82.5)	
Humiliated by her husband in front of others	Yes	7 (6.5)	61 (18.8)	69 (15.9)	0.001
	No	101 (93.5)	264 (81.2)	365 (84.1)	
luxury and rivalry woman	Yes	3 (2.8)	43 (13.2)	46 (10.6)	0.001
	No	106 (97.2)	282 (86.8)	388 (89.4)	
Unwarranted and unreasonable expectations of women	Yes	3 (2.8)	42 (12.9)	45 (10.4)	0.001
	No	106 (97.2)	283 (87.1)	389 (89.6)	
Huff consecutive women	Yes	13 (11.9)	93 (28.6)	106 (24.4)	0.000
	No	96 (88.1)	232 (71.4)	328 (75.6)	
Family intervention in the lives of women	Yes	5 (4.6)	47 (14.5)	52 (12)	0.003
	No	104 (95.4)	278 (85.5)	382 (88)	
Woman's physical or mental illness	Yes	1 (0.9)	17 (5.2)	18 (4.1)	0.036
	No	108 (99.1)	308 (94.8)	416 (95.9)	
Listening to the woman's family	Yes	5 (4.6)	57 (17.5)	62 (14.3)	0.000
	No	104 (95.4)	268 (82.5)	372 (85.7)	

**Table 4:** The relationship between women's mental health and characteristics of marriage in divorce applicant women

**Features marriage**		**Mental Health**			**p.value**
		**Less than 18**	**More than 18**	**Total**	
Consanguinity with partner	Yes	48 (44)	118 (36.3)	166 (38.2)	0.093
	No	61 (56)	207 (63.7)	268 (61.8)	
Family consent	Yes	93 (85.3)	248 (76.3)	341 (78.6)	0.030
	No	16 (14.7)	77 (23.7)	93 (21.4)	
Forced marriage	Yes	26 (24.8)	77 (23.7)	103 (23.7)	0.450
	No	83 (75.2)	248 (76.3)	331 (76.3)	
Previous research conducted	Yes	64 (58.7)	142 (43.7)	206 (52.5)	0.005
	No	45 (41.3)	183 (56.3)	228 (47.5)	
Previous familiarization	Yes	38 (34.9)	118 (36.3)	156 (35.9)	0.440
	No	71 (65.1)	207 (63.7)	278 (64.1)	
Feeling of inferiority in life	Yes	69 (63.3)	161 (49.5)	230 (53)	0.008
	No	40 (36.7)	164 (50.5)	204 (47)	
the method of introduction with partner	Living environment	31 (28.4)	76 (23.4)	107 (24.7)	0.401
	Workplace	6 (5.5)	20 (6.2)	26 (6)	
	University	3 (2.8)	11 (3.4)	14 (3.2)	
	Family	63 (57.8)	190 (58.5)	253 (58.3)	
	Accidental	2 (1.8)	6 (1.8)	8 (1.8)	
	Phone or the Internet	4 (3.7)	22 (6.7)	26 (6)	
During the nomination period (month)	< 6	68 (62.4)	224 (68.9)	292 (67.3)	0.235
	6-12	31 (28.4)	64 (19.7)	95 (21.9)	
	13-24	8 (7.4)	25 (7.7)	33 (7.6)	
	> 24	2 (1.8)	12 (3.7)	14 (3.2)	

Chi-square test revealed a significant association between mental health score category and the previous research about men (p = 0.005), the main family satisfaction with marriage (p = 0.030) and lack of a sense of self-confidence in life (p = 0.008) (Table 4).

More than half of the cases of divorce (53.7 %) were based on mutual agreement. The frequency of mental health scores below 18 was higher found to be among women who chose to divorce based on mutual agreement (59.6 %). On the other hand mental scores below 18 were least frequently observed in the group where divorce was applied by husband (12.8 %). Decision to divorce was made 6 month prior to divorce in 148 (34.1 %) of subjects. The chi-square test revealed a significant association between history of divorce in the family (p = 0.001) and friends (p = 0.001) with mental health score category. More than half of the subjects (50.7%) agree with divorce for their children. There was a significant correlation between the family opinion about divorce and mental health score category (p = 0.001) (Table 5).

**Table 5:** The relationship between women's mental health and characteristics of divorce in divorce applicant women

**Features marriage**		**Mental Health**			**p.value**
		**Less than 18**	**More than 18**	**Total**	
Applicants for divorce	Adaptive	65 (59.6)	168 (51.7)	233 (55.7)	0.310
	Women	30 (27.6)	100 (30.8)	130 (30)	
	Men	14 (12.8)	57 (17.5)	71 (16.3)	
Time of divorce application	Before starting shared living	7 (6.4)	28 (8.6)	35 (8.1)	0.308
	After starting shared living	102 (93.6)	297 (91.4)	399 (91.9)	
Divorce in the family and relatives	Yes	34 (31.2)	163 (50.2)	197 (45.4)	0.001
	No	75 (68.8)	162 (49.8)	237 (54.6)	
Divorce in friends	Yes	17 (15.6)	107 (32.9)	124 (28.6)	0.001
	No	92 (84.4)	218 (67.1)	310 (71.4)	
Length of decision to divorce (month)	< 6	39 (35.8)	109 (33.5)	148 (34.1)	0.149
	6-12	35 (32.1)	80 (24.6)	115 (26.5)	
	> 12	35 (32.1)	136 (41.8)	171 (39.4)	
Family opinion about divorce	Agreeable	70 (64.2)	150 (46.2)	220 (50.7)	0.001
	Disagreement	12 (11)	83 (25.5)	95 (21.9)	
	Disinterested	27 (24.8)	92 (28.3)	119 (27.4)	

## Discussion

This study examined the factors affecting the mental health of women seeking divorce referred to Ahvaz Legal Medicine department during 2011.In this section, the results of the research are based on the research hypotheses will be discussed and will be compared with the results of other studies.

In relation to the first hypothesis of the study "Demographic characteristics associated with mental health of divorce applicant women referred to Ahvaz Legal Medicine department", the mean age of study participants was 30.7±8.4 years. Almost half of women (48.8%) in the age range of 30-20 years living together for less than 5 years (46.1%). The highest and lowest frequency of divorce by age group was in 29-20 years and age group under 20 years respectively. There was no correlation between mental health and age, duration of divorce from marriage. In the study by Yousefi in Saghez city (2012) 65% of divorce cases happened between 1 and 5 years after marriage and 62% of divorces occurred between 25 and 30 years old (17). In the study by Honariyan and Younesi in Tehran (2012) maximum number of divorce were found to occur between the age of 20 and 30 years and in the first five years after marriage (18). These findings were in line with the findings of our study In contrast the study by Ghotbi et al. in Dolat -Abad (2004) showed that the highest prevalence of divorce was found in the age 26-35 year old group and the average age of women was 24 years old at the time of divorce (19). The difference between this study and the study by Ghotbi et al. might be due to the difference in defining age groups. Fatehi Dehaghani reported that half of their divorced subjects divorced within the first 5 years from marriage and that determined this period as the most critical time for the occurrence of divorce, which was consistent with the findings of our study (20).

Almost half of the women seeking divorce (45.2%) had no children may be due to divorce during the early years after marriage while 8.4 % of women had more than 3 children. There was no significant relationship between number of children and mental health of divorce applicant women. But normal mental health was observed to be more prevalent in women without children (52.3%) and least frequent in women more than 3 children (2/8), respectively. This may be due to concerns about the fate of children and the feeling of guilt for their children. Bolhori et al. reprted that 7.6% of Tehrani women who applied for divorce had more than 3 children (21). This finding was in consistent with our study. Yazdi et al. (2009) suggested that having a child can reduce anxiety, depression and stress in women and also having children can cause of encouragement in their mothers (22). That was not consistent with the findings of our study. The educational level of 42.2% of women and 48.8% of their husbands was at guidance school and lower. There was a significant association between education levels of woman her partner and scores of women's mental health. It seems that with increasing educational level of women, their mental health improves. In other words, in women with higher the education level of their spouses, the reaction to life situations will be associated with more appropriate feelings and they are more able to solve family problems. Thus, literacy is an awareness factor and illiteracy and semi literacy can result in higher proportion of divorced (23). In the study by Bolhari in Tehran (2012) education level of 49.6% of applicants was reported to be high school diploma or lower (21). This finding was also is consistent with our study.

Almost three-quarters of the women were housewives and more than half of their husbands were self-employed. There was a significant association between women and their husbands’ job with mental health of divorce applicant women. But this relationship was not observed between the incomes of men and women and mental health of divorce applicant women. The best mental health status was observed in employee women, which seems to be due to better and stable salary and higher education level. In this study, it was observed that mental health scores increased with increasing income levels of husband, which was inconsistent with their of jobs and education status. It seems that the cause of this difference is due to the incorrect reporting of husband income. 

Mandemakers et al. (2010) examined the impact of family history on the divorce related stress and the found that some of the family characteristics have a significant correlation with psychological distress (24). Based on their findings 61/8% of participants lived in families with more than four children, 38.2% of couples had consanguineous relationships, 78.6% married with family consent, 23.7% were forced to marry and 47.5% of the divorcees’ parents did not perform enough research on their child marriage. Besides, 61.4 % of subjects had no history of previous acquaintance with their spouses and 58.3% of subjects were introduced by their family members.

Choice of spouse and marriage is one of the most difficult decisions in lifeand there is no doubt that, as in all aspects of life, the family has a great role in this matter.Many studies indicated disorders in marriage isan important factor in hospital admission for mental disorders (25). In previous studies on men, a significant relationship was reported between woman mental health and family agreement with marriage and sense of inferiority in life in husband.

In this study 30% of women chose to divorce themselves. In 45.3% of women have a history of divorce in family and 28.4 % of them had positive cases of divorce in their friends. In 34.1% of the cases emotional divorce has occurred less than six months prior to decision to divorce. More than half of the families agreed with divorce of their children. There was no significant correlation between requesting divorce and mental health of divorce applicant women but the highest frequency in women with mental health was observed in those who divorced based on mutual agreement (59.6%) and the least frequency of women with normal mental health was observed in women whose husbands applied for divorce (12.8%). Also there was a significant relationship between mental health of divorce applicant women and history of divorce in the families and friends. Overall, it has been shown that the experience of divorce is much more difficult for someone who does not initiate the divorce process compared to the one who starts the divorce process. Research shows that a person who is not the initiator of divorce suffers from guilt, rejection, and disability in life (22). In Australia, Hewitt et al. (2010) stated that the consequences of divorce are less severe in individuals who themselves applied for divorce or divorced by mutual consent (12). This finding is consistent with our study.

The opinion of family, that plays a supportive role in the protection of women after divorce, is an influencing factor on mental health. In this study, a statistically significant association was found between the opinion of family about divorce and the mental health of divorce applicant women. Amato et al. (2001) reported that presence of divorce among parents can legitimize divorce to end the relationship in their children and reduce the commitment to marriage and family in their children. Amato et al. also states that the probability of divorce is higher among children with divorced parents than others (26). Survey results on the consequences of emotional divorce show that the types of pressures and problems of emotionally divorce are different in women's lives and some families experienced more severe consequences (27). In this study, no significant correlation was found between the duration of emotional divorce and mental health of divorced women.

In relation to the second hypothesis, which was "relationship between social factors divorce request and mental health among divorce applicant women in legal medicine Ahvaz" there was a significant relationship between husbands addiction to one of the types of drug, extramarital relations with other women and men and acts of delinquency by husband and the mental health of divorce applicant women. Therefore, the second hypothesis is confirmed. The psychological pressure of each of these factors can influence women's mental health. Drug or alcohol addiction among males creates a sense of loss for their wives. Drug addiction destroys the dependency among family members so that normal cooperation between men and women disappears. Also an alcoholic person is unable to accept responsibility in family due to the psychological effects of alcohol and they are always avoiding their life positions (28). 

The third hypothesis of the research was to identify the "relationship between cultural factors of divorce request and mental health among divorce applicant women in legal medicine Ahvaz". There no significant association was found between none of the cultural factors influencing divorce and mental health of divorce applicant women. Therefore, the third hypothesis is not confirmed.

The fourth hypothesis of this study was to identify "relationship between economic influencing factors of divorce request and mental health among divorce applicant women in legal medicine Ahvaz". No significant relationship was observed between none of the economic factors affecting divorce with the mental health of divorce applicant women. Furthermore, no significant correlation was found between the total score of economic factors that influence divorce and the mental health of divorce applicant women. Therefore, the fourth hypothesis was not confirmed.

The fifth hypothesis in relation to "relationship between life skills and communication factors divorce request and mental health among divorce applicant women in legal medicine Ahvaz", there was significant relationship between some of the factors related to life skills and communication (excuses and blame, mistrust and suspicion, lack of understanding life, insufficient knowledge of the husband before marriage and disrespect for women by male family) with mental health of divorce applicant women. So the fifth hypothesis is not confirmed. In the present study 

The Sixth hypothesis of this study was to identify the "relationship between family factors that influence divorce request and mental health among divorce applicant women in legal medicine Ahvaz". There was a significant relationship between mental health of divorce applicant women and leaving the family without a reasonable excuse by partner, Huff sequential partner, interference of the family of the spouse on the divorcee’s life, disputes between two families, lack of cooperation in home affairs and life by husband and being forced to divorce in order to marry another person. But there was no significant correlation between the total scores of family factors and the mental health of divorce applicant women. Therefore, the Sixth hypothesis was not confirmed.

The Seventh hypothesis of this study was to identify the "relationship between individual and personal factors that influence divorce request and mental health among divorce applicant women in legal medicine Ahvaz". There was a significant relationship between physical abuse and being beaten, verbal and psychological abuse, temporary or permanent remarriage of partner and mental health of divorce applicant women. There was a significant correlation between the total scores of individual and personal factors and mental health of divorce applicant women. Therefore, the seventh hypothesis was confirmed.

Khaghani Fard states a significant relationship between violence against women and mental health and that the violence by men against women, the lower the mental health of women (29). In a study by Moezi in Chahar Mahal Bakhtiari on the psychological effects of domestic violence and violence against women violence against women was found to be one of the harmful factors that affect women's mental health (30).

## Conclusion

Society and politicians are expected to provide measures to effectively identify the economic, social, family and individual causes of divorce and to make correct decision plan to reduce these factors. Although the present study provides valuable data on the factors that influence divorce application, cross-sectional studies are not capable to show how marital relations and marriage situations can affect mental health. Furthermore, there are factors before and after divorce that can affect mental health, for this purpose, a longitudinal study is recommended to investigate the relationship between factors affecting women's divorce and mental health. Due to the fact that the prevalence of divorce and mental health are to some extent influenced by geographical and cultural factors, it is proposed that similar studies be performed in the regions with different cultural and geographical characteristics. The result of this study showed that personality and family traits can be predictors of mental health status. The findings indicate the need to focus more on the youth who want to get married and their families. It is necessary to take into account factors influencing the marriage including coordination between family cultures, education level of spouses, acceptable physical state of a partner from the point of view of the other, couples intention to fully understand each other and considering the financial situation and income of couples in order to prevent divorce, which has a destructive effect on individual and society.

The findings of this study provide information about divorce applicant women; we cannot judge the other women. Another research limitation that maybe influencing in results of study is cultural factors. Therefore, further study in difference society could provide comprehensive information in this area.

## References

[1] Kamly M (2007). Descriptive Study Of The Causes And Risk Factors Of Social Vulnerability For Divorce In Iran, According To The Statistics And Records. Journal Of Danesh-E-Entezami.

[2] Banaeian SH, Parvin N, Kazemian A (2005). The Relationship Between Mental Health And Marital Satisfaction Married Women. Scientific Journal Of Hmadan Nursing&Midwifery.

[3] Naimi M (2011). Family Interaction With Satellite And Incidence Of The Phenomenon Of Divorce-(Case Study City Of Gorgan). Journal Specialized Sociology Youth Studies.

[4] Kalantari A, Roshanfeker P, Javaheri C (2011). Consequences Of The Divorce Systematic Review Research With Emphasis On Gender Issues In Iran. Journal Women In Development & Politics.

[5] Akhavan Tafti M (2002). Divorce Stages And Aftermaths. Journal Womens Studies.

[6] Robbers SC, Bartels M, Van Beijsterveldt CE, Verhulst FC, Huizink AC, Boomsma DI (2011). Pre-Divorce Problems In 3-Year-Olds: A Prospective Study In Boys And Girls. Soc Psychiatry Psychiatr Epidemiol..

[7] Ghotbe M, Holakouee Naeini K, The Jazayeri, Rahimi A (2004). Evaluation Of Divorce And It's Factors In Divorced Person Lived In Doulat Abad (Tehran). Journal Social Welfare Quarterly.

[8] Yousefi N (2012). Comparison Of The Effectiveness Of Family Therapy Based On Schema Therapy Andbowen’s Emotional System Therapy On The Early Maladaptive Schema Amongdivorce Applicant Clients. The Quarterly Journal Of Fundamentals Of Mental Health.

[9] National Population Register (2011). IRAN Statistical Yearbook.

[10] Hafarean L, Aghaei A, Kajbaf MB, Kamkar M (2009). The Compare Between Divorced Women's Quality Of Life And Undivorced Women In Shiraz City And The Study Of Relationship Between Demographic Variables Of Divorced Women And Their Quality Of Life. Journal Of Knowledge And Research In Applied Psychology.

[11] Gholami A, Bshlydh K (2011). Effects Of Spiritual Healing On The Mental Health Of Divorced Women. Journal Of Counseling And Family Therapy.

[12] Hewitt B, Turrell G (2011). Short-Term Functional Health And Wellbeing After Marital Separation: Doesinitiator Status Make Adifference. Am J Epidemiol.

[13] Cohen O, Finzi-Dottan R (2012). Reasons For Divorce And Mental Health Following The Breakup. Journal Of Divorce And Remarriage.

[14] Barron CR (1989). Causal Explanations And Emotional Health Of Women During Divorce. Arch Psychiatr Nurs.

[15] Moshki M, Shahghasemi Z, Delshad Noghabi A, Moslem A (2011). The Survey Of Condition And Related Factors To Divorce From Divorced Couples’ Viewpoint Of Gonabad City In 2009-2010. Ofogh-E-Danesh; Journal Of Gonabad University Of Medical Sciences.

[16] Asgari P, Kalighi Sigaroody E, Yoosefian F, Marashian F (2009). The Effectiveness Of Training Prophet Mohammd Practical Educational Methods On The Religious Thinkin The Religious Beliefs And Attitude And The Mental Health Of High School Girl In Ahvaz City. Journal Knowledge&Research In Applied Psycholog.

[17] Yousefi N (2012). Comparison Of The Effectiveness Of Family Therapy Based On Schema Therapy And Bowen’s Emotional System Therapy On The Early Maladaptive Schema Among Divorce Applicant Clients. Journal Of Fundamentals Of Mental Health.

[18] Honarian M, Younisy J (2011). The Study Of Divorce Reasons In Tehran Family Courts. Journal Of Clinical Psychology Studies.

[19] Ghotbi M, Holakouee K, Jazayeri A, Rahimi A (2004). Evaluation Of Divorce And It's Factors In Divorced Person Lived In Doulat Abad (Tehran). Journal Social Welfare Quarterly.

[20] Fatehi Dehaghani A, Nazari AM (2011). Sociological Analysis Of Factors Contributing To Couples' Tendency Toward Divorce In Isfahan Province. Journal Societal Security Studies.

[21] Bolhari J, Ramezanzadeh F, Abedininia N, Naghizadeh MM, Pahlavani H, Saberi M (2012). The Survey Of Divorce Incidence In Divorce Applicants In Tehran. Journal Of Family And Reproductive Health.

[22] Yazd Khasti H, Mansori N, Zadeh Mohamadi A, Ahmad Abadi Z (2008). The Relation Of Inclination And Guilt Feeling Of Divorce On Stress, Depression And Anxiety Of Those Are To Divorce In Esfahan And Arak. Journal Of Family Research.

[23] Shirzad A, Kazemi Far AM (2004). Epidemiologic Study Of Divorced Couples Are Referred To Legal Medicine The Department Of In Hamadan In 2000. Journal Of Forensic Medicine.

[24] Mandemakers J, Monden CWS, Kalmijn M (2010). Are The Effects Of Divorce On Psychological Distress Modified By Family Background?. Advances In Life Course Research.

[25] Hosseinian S, Yazdi M, Jasbay M (2007). Relationship Between The Health Of Family And Marital Satisfaction Teacher Education Karaj City. Educational and Psychological Research.

[26] Amato Paul R, Deboer Danelle D (2001). The Transmission Of Marital Instability Across Generations: Relationship Skills Or Commitment To Marriage?. Journal Of Marriage And Family.

[27] Bastani S, Golzari M, Rowshani SH (2011). Emotional Divorce And Strategies To Face It. Journal Of Family Research.

[28] Chayat Ghiasi P, Moein L, Rosta L (2010). Study Of Social Causes Of Tendency To Divorce Among Women Referring To Shiraz Family Court. Journal Of Women And Society.

[29] Khaghani Fard M (2011). Investigate The Role Violence Against Women Gender Socialization And Social Capital Tehran Predict Mental Health Of Women. Journal Of Women’s Studies.

[30] Moezi M, Azame M, Shakeri M, Pur Haider B (2008). Spouse Abuse And Its Relationship To Mental Health - Chahar Mahal And Bakhtiari 2006. Journal Of Ilam University Of Medical Sciences.

